# Probiotic *Escherichia coli* Nissle 1917-derived outer membrane vesicles enhance immunomodulation and antimicrobial activity in RAW264.7 macrophages

**DOI:** 10.1186/s12866-020-01953-x

**Published:** 2020-08-27

**Authors:** Rujiu Hu, Hua Lin, Jing Li, Yuezhen Zhao, Mimi Wang, Xiaoqin Sun, Yuna Min, Yupeng Gao, Mingming Yang

**Affiliations:** 1 College of Animal Science and Technology, Northwest A&F University, Yangling, 712100 Shaanxi China; 2 Department of Animal Engineering, Yangling Vocation and Technical Colleg, Yangling, 712100 Shaanxi China

**Keywords:** *Escherichia coli* Nissle 1917, Probiotics, Outer membrane vesicles, Extracellular vesicle, Microbiota-host communication, Macrophages

## Abstract

**Background:**

Probiotic *Escherichia coli* Nissle 1917 (EcN) has been widely studied for the treatment of intestinal inflammatory diseases and infectious diarrhea, but the mechanisms by which they communicate with the host are not well-known. Outer membrane vesicles (OMVs) are produced by Gram-negative bacteria and deliver microbial molecules to distant target cells in the host, which play a very important role in mediating bacteria-host communication. Here, we aimed to investigate whether EcN-derived OMVs (EcN_OMVs) could mediate immune regulation in macrophages.

**Results:**

In this study, after the characterization of EcN_OMVs using electron microscopy, nanoparticle tracking and proteomic analyses, we demonstrated by confocal fluorescence microscopy that EcN_OMVs could be internalized by RAW 264.7 macrophages. Stimulation with EcN_OMVs at appropriate concentrations promoted proliferation, immune-related enzymatic activities and phagocytic functions of RAW264.7 cells. Moreover, EcN_OMVs induced more anti-inflammatory responses (IL-10) than pro-inflammatory responses (IL-6 and TNF-α) in vitro, and also modulated the production of Th1-polarizing cytokine (IL-12) and Th2-polarizing cytokine (IL-4). Treatments with EcN_OMVs effectively improved the antibacterial activity of RAW 264.7 macrophages.

**Conclusions:**

These findings indicated that EcN_OMVs could modulate the functions of the host immune cells, which will enrich the existing body of knowledge of EVs as an important mechanism for the communication of probiotics with their hosts.

## Background

It is increasingly recognized that probiotics play an important role in maintaining intestinal health and regulating immune function in humans and animals [1]. Many probiotics have been developed as pharmaceutical products or dietary supplements to treat intestinal dysfunctions and diseases, such as diarrhea, irritable bowel syndrome and inflammatory bowel disease (IBD) [2, 3]. A large number of in vitro and in vivo studies have indicated that the probiotic-mediated effects are mostly achieved through indirect ways, such as strengthening the intestinal epithelial barrier, regulating the immune system, and competing with pathogens for adhesion to mucosa [4, 5]. Growing attention has been paid to elucidate the molecular mechanisms of communication between probiotics and their hosts.

*Escherichia coli* strain Nissle 1917 (EcN) is a well-known probiotic isolated by Alfred Nissle from the faeces of a soldier who was not infected during the outbreak of Shigellosis [6]. EcN is not pathogenic due to the lack of virulence factor genes in its genome when compared to pathogenic *E. coli* [7]. It is known that EcN can be well colonized in the human intestinal tract and modulates intestinal homeostasis and microflora balance [8, 9]. EcN has been developed as a microbial product under the brand name Mutaflor, which is widely distributed in Central Europe to treat intestinal inflammatory diseases and infectious diarrhea [2]. Numerous studies have confirmed the immunomodulatory mechanisms of EcN, including induction of antimicrobial peptide expression, increase of immunoglobulin A and mucin secretion, enhancement of the intestinal barrier and promotion of anti-inflammatory immune response [10–12].

Although many publications have revealed the EcN-mediated effects, it is still unclear how the EcN establishes the crosstalk with their hosts. Almost all Gram-negative bacteria and some Gram-positive bacteria produce nano-meter membrane vesicles into the extracellular space, which are called extracellular vesicles (EVs) [13]. EVs secreted by Gram-negative bacteria are derived from the outer membrane and thus termed as outer membrane vesicles (OMVs). These vesicles are characterized as spherical bilayered phospholipid structures with diameters between 20 and 200 nm [14]. Purified OMVs contain a variety of bioactive molecules, such as cell-wall components, periplasmic proteins and bacterial nucleic acids, and recently have been considered as a key intercellular communication platform [13]. OMVs can deliver these components in a stable and efficient manner directly to host cells and affect their biological functions, including inducing pathogenesis, modulating immune response and signaling [14, 15]. Many studies in the last few decades on pathogens, such as *Vibrio cholerae* [16], *Staphylococcus aureus* [17] and *Salmonella* [18], have indicated that OMVs cause cytotoxic responses of target cells by delivering virulence factors. Recently, several studies have found that OMVs derived from commensal bacteria or probiotics also play a key role in microbiota-host interactions. OMVs secreted by *Bacteroides fragilis* [19] and *Akkermansia muciniphila* [20], two species of symbiotic bacteria in the human intestinal, were found to deliver immunomodulatory molecules to intestinal immune cells and induce anti-inflammatory immune responses. *Bifidobacterium* is a widely used Gram-positive probiotic, which produces EVs as mediators to activate intestinal immune cells [21].

Recently, vesicular proteins associated with adhesion and immune regulation were identified from EcN-derived OMVs (EcN_OMVs), indicating that the secretory vesicles have the potential to modulate the interaction of EcN with its host [22]. Moreover, Fábrega et.al and Alvarez et.al demonstrated that EcN_OMVs are involved in the induction of immune and defense responses in the intestinal mucosa barrier [23, 24]. In this study, we further investigated whether the EcN_OMVs mediate the regulations of host immune cells. We analyzed the proteome of EcN_OMVs and then evaluated the regulation of the immune responses and antimicrobial activities by mouse macrophage RAW264.7 cells upon stimulation with EcN_OMVs in an in vitro model. This finding will enrich the existing body of knowledge of EVs as an important mechanism for the communication of probiotics with their hosts.

## Results

### Preparation and proteomic analysis of EcN_OMVs

**Fig. 1:**
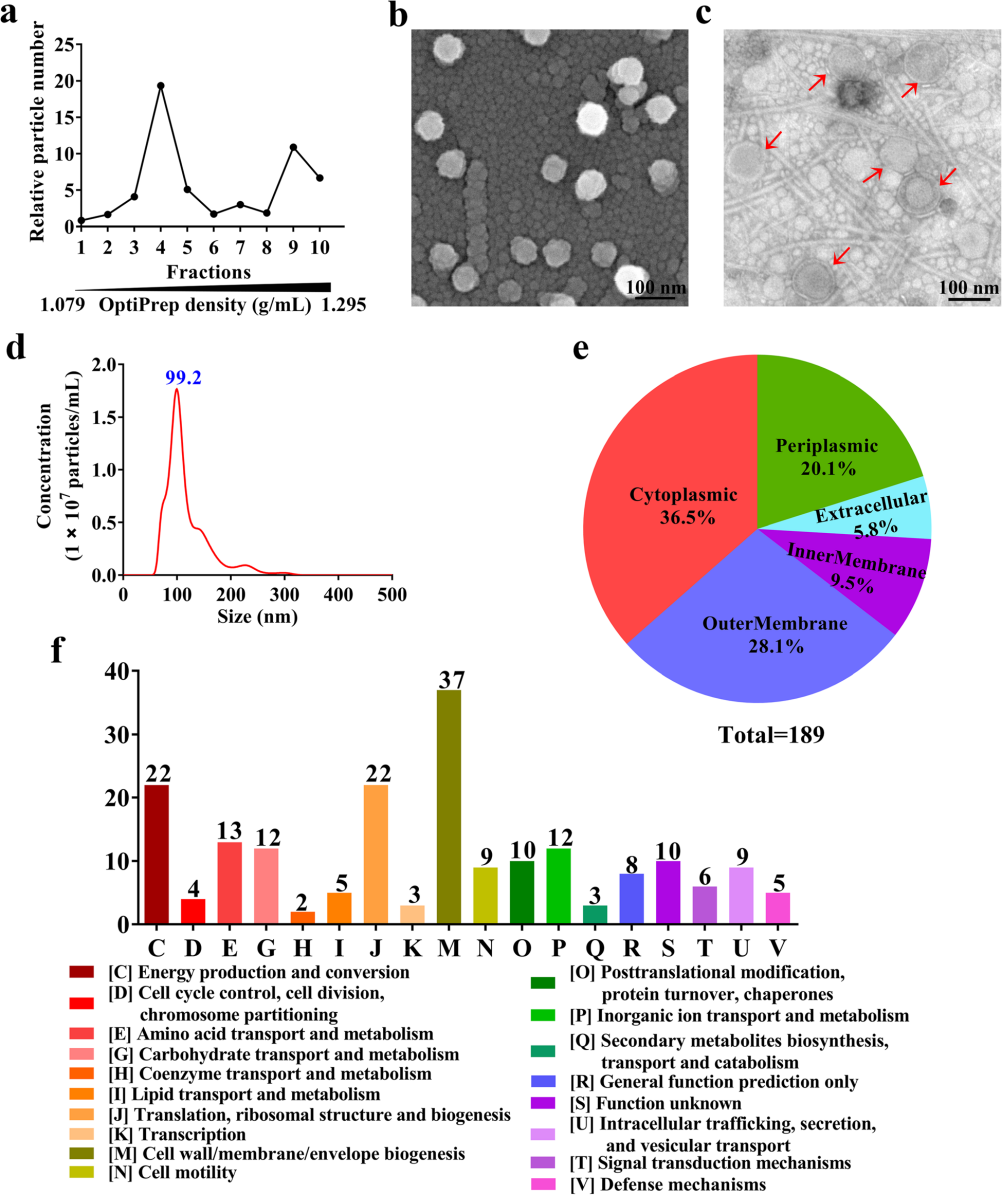
Visualization and characterization of OMVs derived from probiotic *Escherichia coli* Nissle 1917 (EcN_OMVs). **a** The particle numbers of the resulting fractions (1–10) after EcN_OMVs purification by Optiprep density gradient ultracentrifugation. Representative scanning electron micrograph (**b**) and transmission electron micrograph (**c**) of purified EcN_OMVs (indicated by red arrows). **d** Size distribution and concentration of these vesicles determined by nanoparticle tracking analysis. **e** Subcellular localizations of EcN_OMVs proteins identified by proteomic analysis. **f** Identified proteins according to Clusters of Orthologous Groups of proteins (COG)

**Fig. 2:**
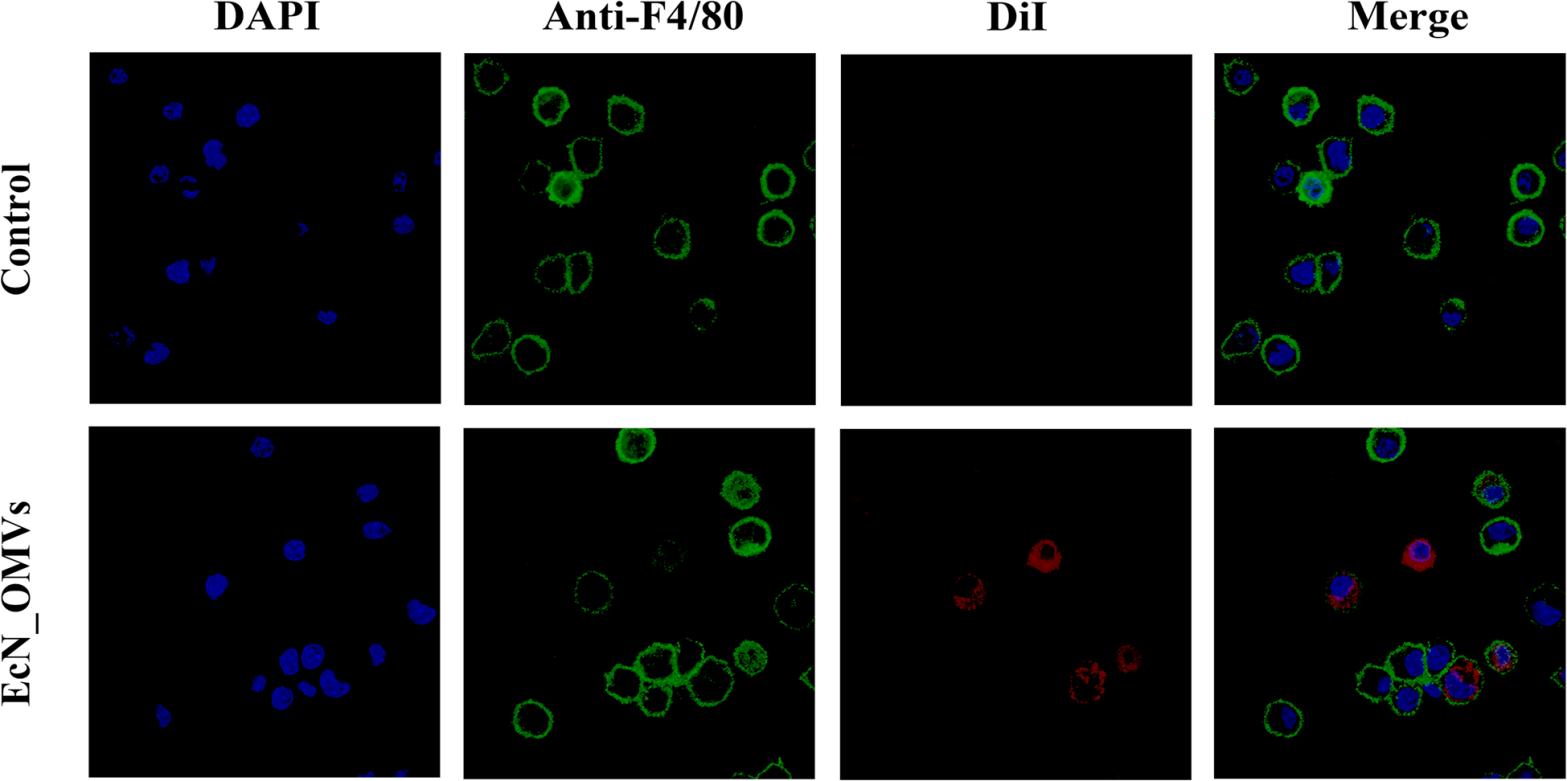
Internalization of EcN_OMVs by RAW264.7 macrophages. DiI-labeled EcN_OMVs (red signal) were incubated with RAW264.7 cells for 16 h at 37 °C. The cell membrane was visualized by immunostaining with anti-F4/80 antibody (5 μg/mL), the macrophage marker, followed by Dylight Fluor-conjugated Goat Anti-Rat IgG (green signal), and the cell nuclei were stained with DAPI (blue signal). The samples were observed using the High-speed spinning-disk confocal microscope

Purified EcN_OMVs were obtained from the culture supernatant of EcN using a series of filtration and centrifugation steps. After the OptiPrep density gradient ultracentrifugation, we found a large number of particles in fractions 4–6 and 9–10 using the nanoparticle tracking analysis (NTA) (Fig. 1a). The density ranges for these fractions were from 1.127 to 1.175 g/mL and 1.271 to 1.295 g/mL, respectively, which is in accordance with the previous results [25]. These vesicles-containing fractions were pooled and subsequently visualized using scanning electron microscopy (Fig. 1b) and transmission electron microscopy (Fig. 1c). These micrographs revealed that these vesicles were spherical particles with a size range of 50–150 nm. This finding was confirmed by the result from NTA characterization of EcN_OMVs, which showed a peak at 99.2 nm for the size distribution of these vesicles (Fig. 1d). Furthermore, 189 proteins were identified by the LC-MS/MS analysis, and their subcellular localizations are presented in Fig. 2e. The subcellular localization of these identified proteins showed that 36.5% (69), 28.0% (53), 20.1% (38) and 9.5% (18) belonged to the cytoplasm, outer membrane, periplasm and inner membrane, respectively. According to the functional classification by the Clusters of Orthologous Groups of proteins (COG), most of these proteins were significantly classified into metabolism, transporter activity, translation and transcription (Fig. 2d). All proteins identified in EcN_OMVs, their subcellular localization and COG functions are presented in **Table** **S1**. We found that many vesicular proteins, mainly of outer membrane origin, might contribute to the probiotic behavior of EcN, including intestinal adhesion and colonization (such as the flagellins: flgA, flgE and flgK), bacterial survival in host niches (such as many proteins associated with transport activities), antimicrobial activities (such as the murein hydrolase: mltA and mltC), and immunomodulation to the host (such as many outer membrane proteins: OmpA, OmpC and OmpF). These multiple probiotic-related proteins identified in EcN_OMVs suggested that they might mediate the effects of this probiotic on immune regulation and disease protection.

### EcN_OMVs taken up by RAW 264.7 macrophages

Previous studies have suggested that OMVs secreted by Gram-negative bacteria can be internalized by the host cell and deliver biological components to regulate the host immune system [14, 23]. In this context, we sought to confirm whether EcN_OMVs could be taken up by macrophages. EcN_OMVs were stained with the red fluorescent lipophilic compound DiI and the cell membranes and nucleus were labeled with anti-F4/80 antibody and DAPI, respectively. After co-incubation of macrophages with DiI-labeled EcN_OMVs for 16 h, EcN_OMVs were observed in the cytoplasm of the cells by confocal fluorescence microscopy, indicating that these vesicles were internalized by RAW 264.7 cells (Fig. 2).

### EcN_OMVs at appropriate concentrations promote the proliferation of RAW264.7 cells

**Fig. 3:**
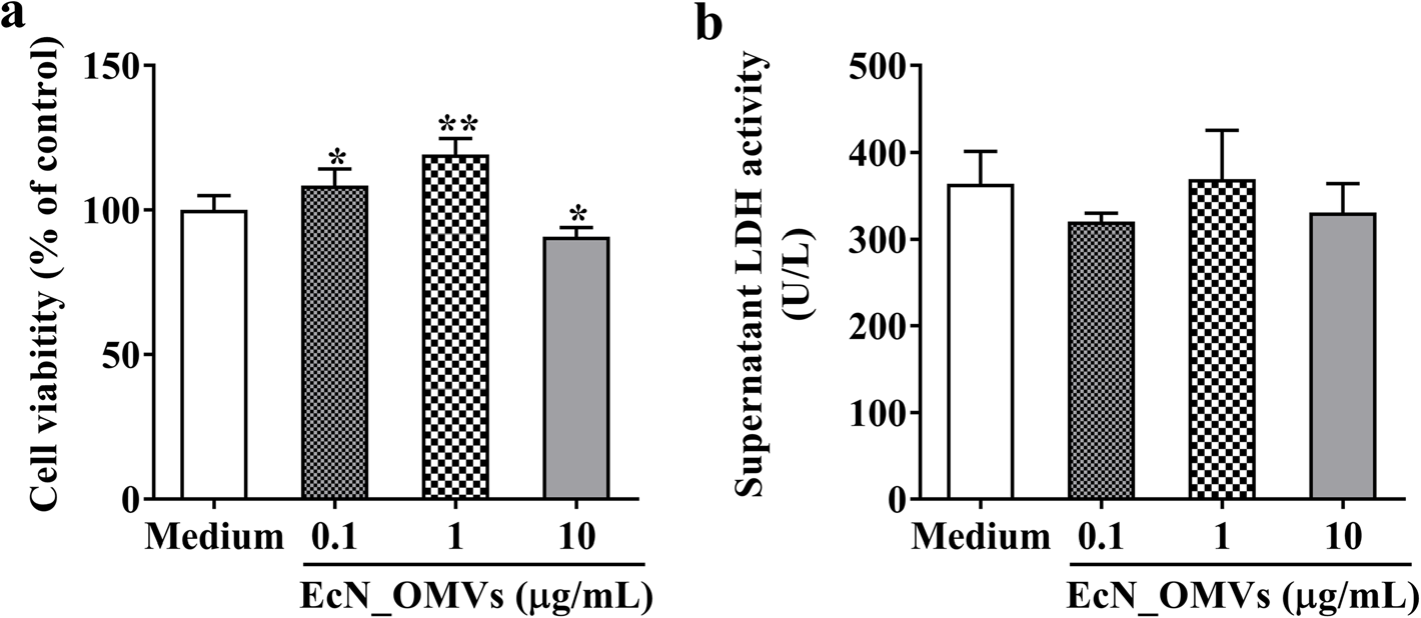
EcN_OMVs at moderate concentrations promote the proliferation of RAW264.7 cells. **a** Viability of RAW264.7 cells at 16 h after exposure to EcN_OMVs at various concentrations. **b** The LDH activity in the cell supernatant at 16 h after exposure to EcN_OMVs at various concentrations. Data are representative of three independent experiments. *P < 0.05; **P < 0.01; versus control

As shown in Fig. 3a, compared with the control group, the cell viability was significantly increased by 8.5 and 19.35% at 16 h after exposure to 0.1 and 1.0 μg/mL of EcN_OMVs, respectively, while significantly decreased by 8.1% at 16 h after exposure to 10 mg/mL of EcN_OMVs, suggesting that the vesicles at moderate concentrations were non-toxic to RAW264.7 cells. Lactate dehydrogenase (LDH) is an endoenzyme in normal living cells, and its activity in the cell supernatant reflects the integrity of the cell membrane. As shown in Fig. 3b, there was no significant difference in LDH activity among the EcN_OMVs-treated groups and the control group, indicating that the concentrations of EcN_OMVs used in this study did not cause cell damage. Together, these findings revealed that EcN_OMVs at appropriate concentrations could promote the proliferation of RAW264.7 cells. The concentration of 1.0 μg/mL was chosen as the final concentration of EcN_OMVs for the subsequent investigation in this study.

### EcN_OMVs improve immune-related enzymatic and phagocytic activities

**Fig. 4:**
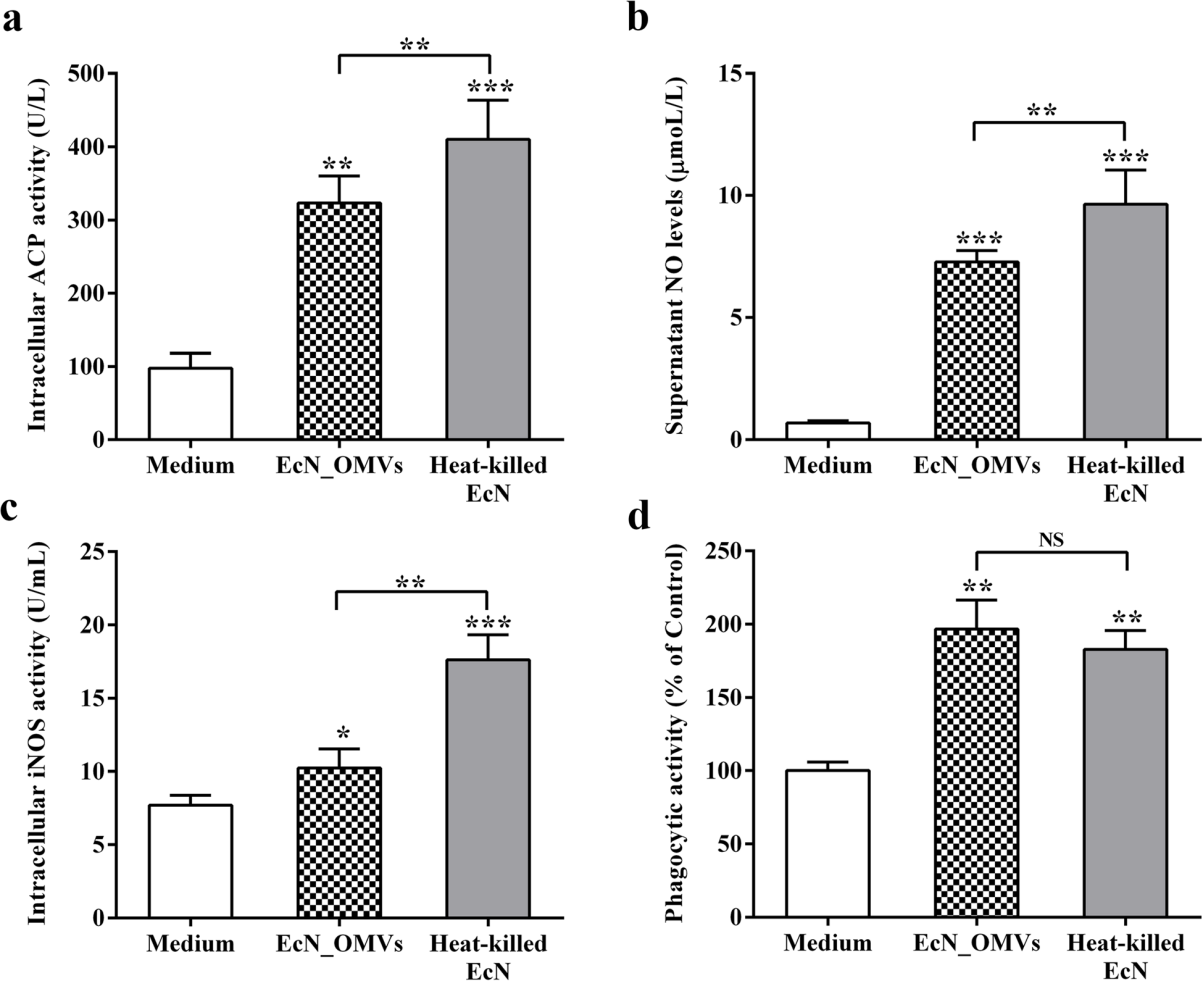
EcN_OMVs modulate immune-related enzymatic and phagocytic activities in RAW264.7 cells. RAW264.7 cells were stimulated with EcN_OMVs and heat-killed EcN for 16 h. After these stimulations, cell supernatants and lysates were collected for examining the following indicators: **a** ACP activity in cell lysates; **b** NO production in cell supernatants; **c** iNOS activity in cell lysates. **d** Phagocytic activity of RAW264.7 cells to FITC-labeled dextran at 16 h after stimulations with EcN_OMVs and heat-killed EcN. Data are representative of three independent experiments. *P < 0.05; **P < 0.01; ***P < 0.001; NS., not significant; versus control

Acid phosphatase (ACP) is associated with the phagocytosis and clearance of exogenous substances by macrophages. The ACP activity was significantly improved when RAW 264.7 cells were stimulated with EcN_OMVs and heat-killed EcN (Fig. 4a), indicating that both these vesicles and heat-killed EcN activated macrophages and enhanced their immune function. NO is an important messenger molecule secreted by macrophages for immune responses. Compared with the control, stimulations with EcN_OMVs and heat-killed EcN significantly induced NO production in the cell culture supernatants (Fig. 4b). The higher activity of inducible nitric oxide synthase (iNOS) found in RAW 264.7 cells after stimulation with EcN_OMVs and heat-killed EcN was well in accordance with the results of NO determination (Fig. 4c). Furthermore, the phagocytic activity of RAW 264.7 cells was significantly improved at 16 h after stimulation with EcN_OMVs and heat-killed EcN (Fig. 4d). Together, these data suggested that EcN_OMVs could activate RAW 264.7 cells and enhance their phagocytosis.

### EcN_OMVs induce immunomodulatory cytokine secretion in RAW264.7 macrophages

**Fig. 5:**
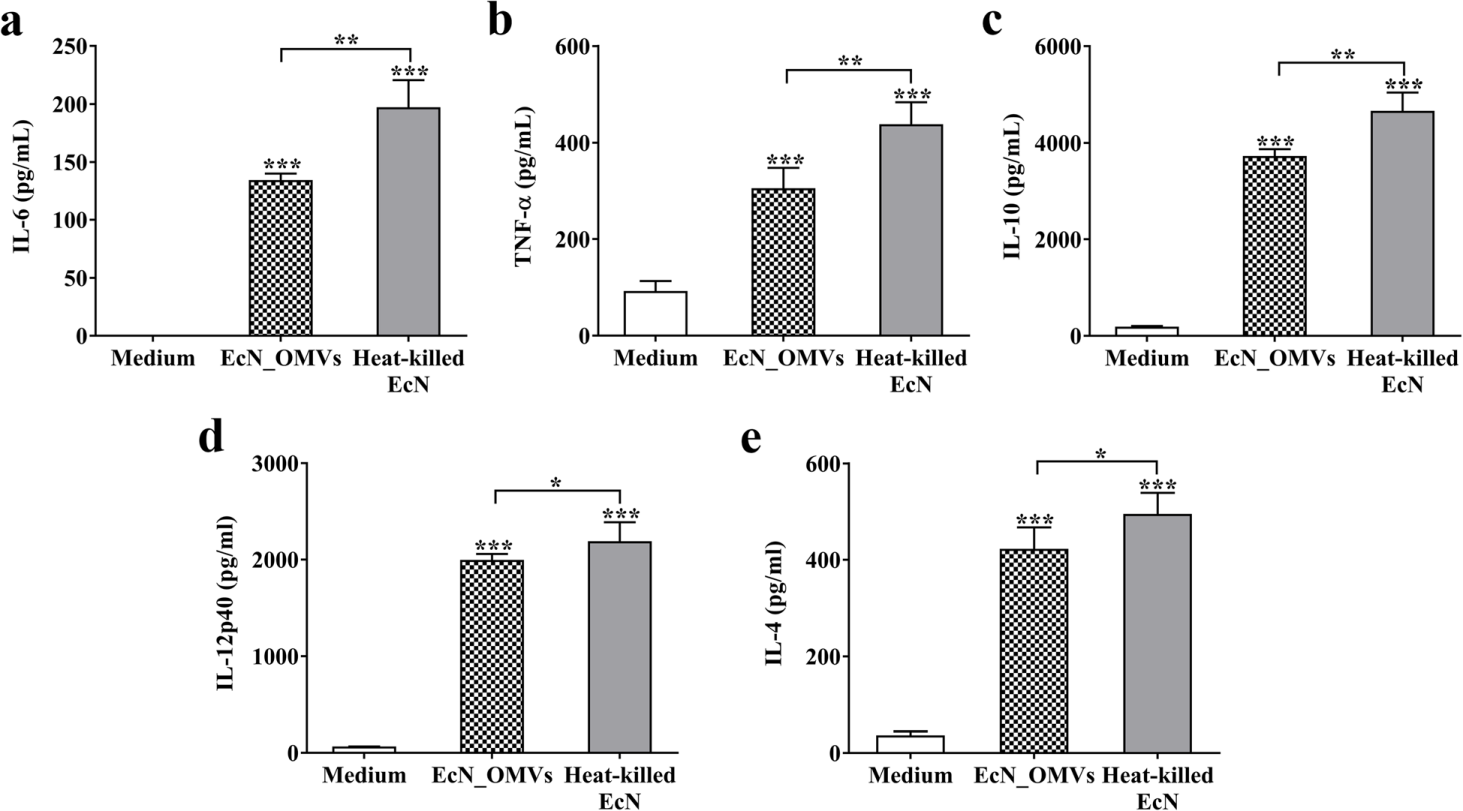
Profile of cytokine secretion by RAW264.7 macrophages stimulated with EcN_OMVs. RAW264.7 cells were stimulated with EcN_OMVs and heat-killed EcN for 16 h. After these stimulations, cell supernatants were collected for determining the following cytokines: **a** IL-6; **b** TNF-α; **c** IL-10; **d** IL-12p40; **e** IL-4. Data are representative of three independent experiments. *P < 0.05; **P < 0.01; ***P < 0.001; versus control

We next evaluated the immunomodulatory effects of EcN_OMVs on RAW 264.7 cells. As illustrated in Fig. 5 a, b and c**,** both EcN_OMVs and heat-killed EcN induced the significant production of pro-inflammatory cytokines (IL-6 and TNF-α) and anti-inflammatory cytokine (IL-10) by RAW 264.7 cells. Compared to the stimulation with heat-killed EcN, EcN_OMVs promoted lower secretion levels of these cytokines. EcN_OMVs triggered higher induction of anti-inflammatory cytokines (ng range) than that of pro-inflammatory cytokines (pg range). Furthermore, EcN_OMVs and heat-killed EcN also efficiently stimulated the secretion levels of IL-12p40 (a representative Th1-polarizing cytokine; Fig. 5d) and IL-4 (a representative Th2-polarizing cytokine; Fig. 5e). These results revealed that EcN_OMVs efficiently induced the immune responses by macrophages and stimulated macrophages to secrete immunomodulatory cytokines.

### EcN_OMVs improve the antibacterial activity of macrophages

**Fig. 6:**
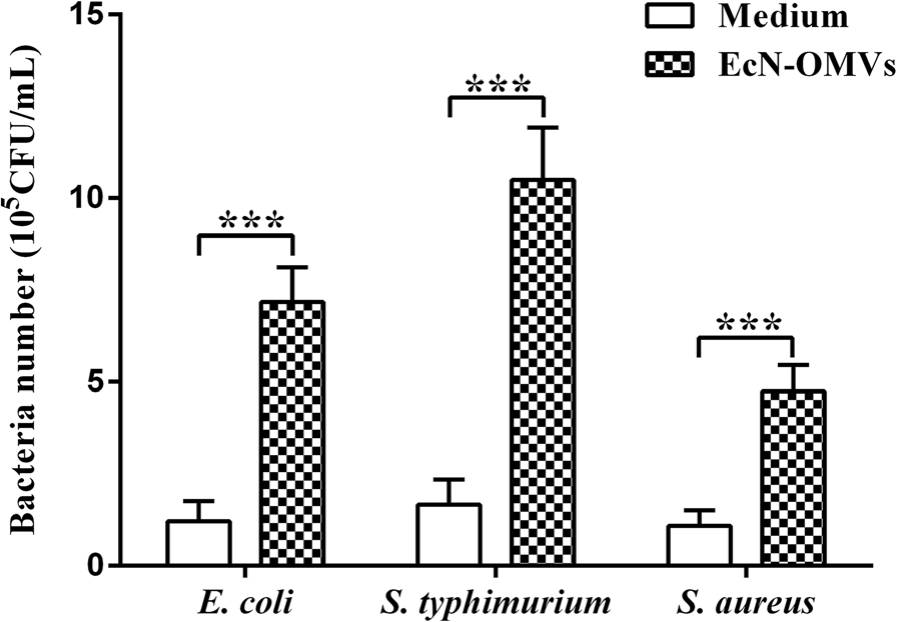
Antimicrobial activity of macrophages stimulated with EcN_OMVs. RAW264.7 cells stimulated with EcN_OMVs were incubated with three bacterial pathogens for 5 h, including two Gram-negative species, *E. coli* CVCC1554 and *S. typhimurium* CVCC3757, and one Gram-positive specie, *S. aureus* CVCC4265. After incubation, the number of viable bacteria in cell lysates was determined using the plate cultivation method. Data are representative of three independent experiments. ***P < 0.001

To evaluate whether EcN_OMVs directly affects the ability of macrophage to fight bacterial infection, we examined the bacteria-killing ability of RAW 264.7 cell stimulated by EcN_OMVs using three bacterial pathogens, including *E. coli* CVCC1554, *S. Typhimurium* CVCC3757, and *S. aureus* CVCC4265. As shown in Fig. 6, after bacterial infections for 5 h, the cells treated with EcN_OMVs showed a stronger bactericidal ability against these three pathogens compared with the control group, indicating that EcN_OMVs enhanced the antibacterial activity of macrophages.

## Discussion

In the last few decades, extensive studies have revealed that gut microbiota plays a very important role in the development and function of the host’s immune system [26]. The dysbiosis of gut microbiota, which can trigger inappropriate immune activation and inflammation in the intestine, is closely associated with several gastrointestinal diseases and particularly with IBD [27]. Administration of probiotics is considered as a promising strategy to restore intestinal microbiota composition and regulate the host immune response [1]. EcN is one of the most widely used probiotics for the treatment of intestinal disorders. Numerous studies have confirmed the therapeutic efficacy of EcN both in murine models of experimental colitis and human IBD [9, 28, 29]. Among the mechanisms by which EcN exerts the beneficial effects, immunomodulation is recognized as a key contributor [30]. EcN-mediated immunomodulatory effects are mainly associated with their ability to induce the development and cytokine secretion of different immune cells in the gut. Besides the direct interaction between the probiotic and immune cells, these observed effects also depend on the release of secreted bacterial mediators [31]. Among the many bacteria-derived factors, EVs play an important role in the communication between microbiota and the host [14, 23], as they can drive the long-distance transport of interior molecules throughout the intracellular compartments in a concentrated, protected and targeted manner [32]. OMVs carry many effector molecules of their parental bacterium such as lipopolysaccharide (LPS), peptidoglycan and DNA, which can be recognized by Toll-like receptor (TLR) 4, Nod-like receptor (NOD) 1/NOD2 and TLR9 in host’s immune cells, respectively [33]. Therefore, OMVs can mediate certain actions of parental bacterium.

Accordingly, it is conceivable that EcN-OMVs could influence the functions of the host’s immune cells. In the present work, we found that EcN-OMVs could effectively be internalized by RAW 264.7 macrophages. This finding is similar to a previous study that showed the uptake of EcN-OMVs by Caco-2 cells [23]. Several studies have identified that bacterial OMVs enter host cells via receptor-mediated pathways or lipid rafts [21, 34]. For macrophages, the uptake of OMVs may also be through random phagocytosis, and the detailed underlying mechanism needs to be further studied. Additionally, the phagocytic activity of macrophage is an important indicator of its immune function. The intracellular enzymes in macrophages, such as ACP and iNOS, are closely related to their phagocytic function [35, 36]. ACP is a hydrolytic enzyme existing in the lysosome of macrophages, which is involved in the digestive function of various lysosomes [37]. iNOS is a key enzyme in the synthesis of NO, which is an important messenger and effector molecule in the defense system of macrophages [36]. Many studies have shown that the activity of ACP and iNOS can be significantly improved after macrophage activation [35, 38]. This study revealed that the intracellular ACP and iNOS activities and the production of NO in the cell culture supernatant were significantly improved after the stimulation of RAW 264.7 cells with EcN_OMVs, indicating that EcN_OMVs activated macrophages and enhance phagocytic functions of these cells. These findings were also confirmed by the phagocytosis assay of FITC-labeled dextran by RAW 264.7 cells and antibacterial activity assay.

It is widely known that immunomodulatory cytokines play a critical role in immune regulation and inflammatory responses. Previous in vitro and in vivo studies have demonstrated that EcN_OMVs could recapitulate anti-inflammatory properties of EcN by modulating cytokine expression and production [23, 31, 39, 40]. Fábrega et.al revealed that EcN_OMVs triggered the expression and secretion of pro-inflammatory cytokines IL-6, IL-8 and TNF-α and anti-inflammatory IL-10 in peripheral blood mononuclear cells [23]. Similarly, EcN_OMVs could elicit the expression of IL-6 and IL-8 in human intestinal epithelial cells in a NOD1 dependent manner, and induce the expression of IL-6, TNF-α, IL-10 and TGF-β in human monocyte-derived dendritic cells [31, 40]. Another study performed in mice model of experimental colitis has shown that treatment of colitic mice with EcN_OMVs could reduce intestinal inflammation by inhibiting the expression of IL-1β, TNF-α and IL-17 and enhancing the expression of IL-10 in colonic tissues [39]. Consistent with these results, the present study showed that stimulation with EcN_OMVs induced the expression of TNF-α, IL-6 and IL-10. Several studies have demonstrated that the up-regulation of pro-inflammatory cytokines by EcN_OMVs was probably due to the presence of LPS or other pattern recognition receptor-ligands, while some yet unidentified vesicular components may induce the activation of IL-10 [23, 41]. In this study, some vesicular proteins known to regulate the host immune response, such as the flagellins, outer membrane proteins and cytoplasmic enzymes, were identified in EcN_OMVs. It should be emphasized that EcN_OMVs increased the production of IL-10 to a higher level compared with that of TNF-α and IL-6. IL-10 produced by macrophages can strongly inhibit the production of pro-inflammatory cytokines in cytokine signaling and plays a critical role in the maintenance of immune response balance [42]. These findings indicated that EcN_OMVs mainly functioned as a modulator of immune homeostasis. Our results are also confirmed by the results of Alvarez et.al showing that EcN_OMVs could enhance the function of the intestinal epithelial barrier ex vivo [24]. Besides, this study also showed that EcN_OMVs activated the expression of Th1-polarizing cytokine (IL-12) and Th2-polarizing cytokine (IL-4) in RAW 264.7 cells. These polarizing cytokines play a key role in regulating the adaptive immune response to host defense [42, 43]. Further in vitro and in vivo studies are required to fully understand the regulation of EcN_OMVs on the adaptive immune response.

As multiple molecules may synergistically contribute to EcN_OMVs-mediated immunomodulation, it is challenging to determine which specific molecules play the most important role. Among the composition of bacterial OMVs, proteins account for the largest proportion and are considered essential components for functions of OMVs [13]. Previously, it has been illustrated that certain proteins isolated from the probiotic-derived EVs exerted a similar effect of the intact EVs. *B. longum* KACC 91563-derived EVs contain a protein ESBP that can induce the beneficial effect of the bacterial EVs [21]. EVs derived from *L. casei* BL23 carry several proteins associated with the probiotic effects of the bacterium, such as p40 and p75 [44]. In this study, several strain-specific proteins were identified in EcN_OMVs by proteomic analysis, such as several subunits (focA, focF, focG and focH) of the specific fimbriae F1C and iron uptake-related proteins (iutA) [22]. F1C-fimbriae is closely related to the biofilm formation and intestinal colonization of EcN [45]. The components of iron acquisition systems may enable this probiotic to gain a competitive advantage against pathogens in host niches [22]. Therefore, these strain-related proteins may be involved in the beneficial effects of the probiotic EcN. Besides, polysaccharides such as LPS are also important components of OMVs. LPS in EcN shows the shortened carbohydrate chain and lacks the repeating units of the O-chain compared to wild-type LPS [46]. Several studies have shown that the truncated LPS may be partially responsible for the anti-inflammatory properties of EcN [41]. Accordingly, it can be inferred that the LPS variant from EcN may confer a relevant contribution to the EcN_OMVs-mediated immunomodulation. Furthermore, probiotics-derived DNA and RNA have also been demonstrated to have inhibitory activity in inflammatory responses. CpG DNA derived from probiotics and commensal bacteria mediates anti-inflammatory responses through TLR 9 signaling [47]. *L. gasseri*-derived RNA suppresses inflammatory responses through a MyD88-dependent signaling pathway [48]. OMVs produced by several commensal bacteria have been shown to contain DNA and RNA. Whether nucleic acids are enclosed in EcN_OMVs and these molecules are involved in the immunomodulatory activities of EcN_OMVs remains to be elucidated.

Although our data present evidence that EcN_OMVs enhanced immunomodulatory effects and antimicrobial function, further studies are needed to arrive at more generalized conclusions. Currently, we have yet to identify these IL-10 inducing anti-inflammatory molecules in EcN_OMVs. Despite the fact that we performed a preliminary proteomic analysis of EcN_OMVs, the role of these vesicular proteins and other non-protein components in regulating the function of macrophages has not been thoroughly validated. Many uncontrollable conditions existing in vivo milieu, such as host lipases that destroy the vesicles, may also influence the interaction between EcN_OMVs and cells. Therefore, in vivo studies are necessary to obtain generalized conclusions. However, notwithstanding these limitations, our results provide support for the biological activity of EcN_OMVs in modulating host immune responses.

## Conclusions

Recent studies have revealed that OMVs released by probiotic EcN strains probably play a very important role in the activation of host immune responses. In this study, we identified vesicular proteins of probiotic EcN-derived OMVs using proteomics, and demonstrated that these vesicles could modulate immune responses and antimicrobial activities in mammalian macrophages in vitro. These results indicate that OMVs could mediate the effects of the probiotic EcN on the host, especially the modulation of intestinal immune homeostasis. Although there were some shortcomings in the present study, we demonstrated EcN_OMVs played an important role in modulating the functions of the host immune cells. This finding will enrich the existing body of knowledge of EVs as an important mechanism for the communication of probiotics with their hosts.

## Methods

### Bacterial strain and growth condition

The probiotic *E. coli* strain Nissle 1917 was purchased from Ardeypharm (GmbH, Herdecke, Germany). The strain was grown at 37 °C in Luria-Bertani (LB) broth with continuous shaking at 180 rpm.

### Macrophage culture

The RAW 264.7 murine macrophage was provided by the Cell Bank of Chinese Academy of Sciences (Shanghai, China). Cells (passages 40–55) and cultivated in complete PRMI-1640 medium (Gibco/Life Technologies Corporation, Grand Island, NY, USA) containing 10% heat-inactivated fetal bovine serum (Zeta-Life, Menlo Park, CA, USA) and Penicillin-Streptomycin solution (100 U/mL of penicillin and 100 μg/mL streptomycin; Sigma-Aldrich, St. Louis, MO, USA) at 37 °C in a 5% CO_2_ atmosphere. The culture medium was exchanged every 24 h and the cells were passaged every 48 h.

### EcN_OMVs isolation and purification

OMVs were obtained from the EcN culture supernatant as described in our previous study [49]. In brief, bacterial cells were grown for 14 h at 37 °C till to late log phase (OD_600_ of 0.9 to 1.0) and were removed by centrifugation at 12,000×g for 20 min at 4 °C. The culture supernatant was passed through a 0.45-μm membrane (JINTENG, Tianjin, China) using a vacuum filtration device (Corning, NY, USA) to remove large particles such as residual bacteria and cellular debris. The filtrate was then concentrated by an Amicon ultrafiltration system (Merck Millipore, Billerica, Massachusetts, USA) with a 100 kDa membrane (Millipore, Billerica, MA, USA). After an additional filtration with a 0.22-μm membrane (Millipore, Billerica, MA, USA), the concentrate was then ultracentrifuged at 150,000×g for 2 h at 4 °C. The EcN_OMVs pellet was washed and resuspended in sterile phosphate buffer saline (PBS; pH 7.4) and then purified by density centrifugation [50]. To remove non-vesicular contamination, the vesicles were purified by discontinuous density centrifugation. The EcN_OMVs fraction at the bottom was mixed with 60% OptiPrep solution (Sigma-Aldrich, St. Louis, MO, USA) to obtain 55% (v/v) OptiPrep solution. A series of 1 mL OptiPrep gradient layers ranging from 5 to 55% (v/v) were overlayed with the vesicle fraction, and centrifuged at 180,000×g (16 h, 4 °C) using a Beckman SW40 Ti swing rotor (Beckman, CA, USA). After centrifugation, each 1 mL fraction was collected from the top of the gradient to the bottom, and the relative particle numbers of each fraction were analyzed by a Nanoparticle Analyser (NanoSight, Malvern, Worchestershire, UK). These vesicles-containing fractions were pooled, diluted in PBS, and then centrifuged (150,000 g, 2 h, 4 °C) to completely remove OptiPrep. The purified EcN_OMVs were uniformly dispersed in sterile PBS followed by filter sterilization with a 0.45-μm membrane (Millipore, Billerica, MA, USA). The EcN_OMVs sample was stored at − 80 °C for future use. The protein quantification of EcN_OMVs was determined by BCA Protein Assay Kit (TaKaRa Bio, Beijing, China).

### Nanoparticle tracking and electron microscopy analyses

Nanoparticle tracking analysis (NTA) was conducted to determine the diameter size and particle number of EcN_OMVs using an NS300 nanoparticle analyzer (Malvern, Worchestershire, UK) [50]. Morphological characteristics of EcN_OMVs were detected with scanning electron microscopy using a Field Emission Scanning Electron Microscope (S-4800, Hitachi, Tokyo, Japan) and transmission electron microscopy using a JEM1011 Electron Microscope at 100 kV (JEOL, Tokyo, Japan), as described previously [51].

### Proteomic analyses

Triplicate biological EcN_EVs samples were sent to Hangzhou PTM Biolabs (Hangzhou, Zhejiang province, China) for proteomic analysis. In brief, proteins (10 μg) of EcN_OMVs were lysed by sonication on ice in lysis buffer (8 M urea, 1% protease inhibitor cocktail, 2 mM EDTA) and separated by 12% SDS-PAGE gel. Major protein bands were extracted from the gel, and then digested trypsin (Promega) at a 1:50 w/w (trypsin to protein) overnight at 37 °C according to an in-gel digestion protocol [52]. The tryptic peptides were processed by the UPLC coupled to tandem mass spectrometry (MS/MS) (LC-MS/MS; Thermo Electron, San Jose, CA, USA) [53]. The obtained MS/MS data were analyzed by the Maxquant search engine (v.1.5.2.8). Database searches were analyzed by using the UniProt database against the EcN genome draft sequence, as described previously [22]. The identified proteins were classified by subcellular localization and Gene Ontology (GO) biological processes according to our previous study [49].

### Visualization of EcN_OMVs uptake by RAW 264.7 macrophage

To evaluate whether EcN_OMVs were taken up by macrophage, they were stained with lipophilic fluorophore dialkylcarbocyanine iodide (DiI; Sigma-Aldrich, St. Louis, MO, USA) as described previously [54]. In brief, the purified vesicles were resuspended with a certain volume PBS in the presence of 1 μM DiI and incubated for 1 h at 37 °C in a water bath. The DiI-labeled EcN_OMVs pellet was obtained by ultracentrifugation at 150, 000×g (2 h, 4 °C). To completely remove the unbound DiI, the pellet was resuspended in PBS and washed three times. After final ultracentrifugation, the DiI-labeled vesicles (3 μg) were resuspended in PBS and then incubated with the RAW 264.7 macrophages for 1 h in a 6-well plate (Corning, NY, USA) at 37 °C in a 5% CO_2_ atmosphere. After the incubation, the cells were collected, washed three times with PBS, fixed with 4% paraformaldehyde for 30 min in PBS, and then penetrated with 0.5% TritonX-100 (Sigma-Aldrich, St. Louis, MO, USA) for 5 min at room temperature. The cells were then blocked with PBS containing 5% bovine serum albumin for 2 h at room temperature. Cell membranes were immunostained with anti-F4/80 Ab (5 μg/mL; Sigma-Aldrich, St. Louis, MO, USA) followed by Dylight Fluor-conjugated Goat Anti-Rat IgG (Abbkine, Redlands, CA, USA) [55]. The cell nucleus was stained with 4, 6-diamidino-2-phenylindole (DAPI) (10 μg/mL; Sigma-Aldrich, St. Louis, MO, USA). Subsequently, the samples were placed over glass slides and visualized by an Andor Revolution XD spinning-disk confocal microscope (Andor Technology, UK) with a 63 × oil immersion objective lens.

### Cytotoxicity of EcN_OMVs

The LDH activity in the cell culture supernatant and cell proliferation activity were detected to evaluate the cytotoxicity of EcN_OMVs. RAW 264.7 cells (1 × 10^5^ cells/well) were grown in 24-well plates, and treated with various concentrations of EcN_OMVs for 16 h. After incubation, the cell culture supernatant was collected for the determination of the LDH activity using the LDH kit (Nanjing Jiancheng Bioengineering Institute, Jiangsu, China). For cell proliferation activity determination, RAW 264.7 cells (2 × 10^4^ cells/well) were grown in 96-well plates, and treated with various concentrations of EcN_OMVs for 6 h followed by completely discarding the cell culture and adding new cell medium for 24-h incubation. After this period, the cell viability was detected by using the CCK-8 cell viability assay kit following the manufacturer’s protocol (Nanjing Jiancheng Bioengineering Institute, Jiangsu, China). Each treatment group was detected in triplicate, and three independent assays were performed.

### Phagocytic activity of RAW 264.7 macrophage

RAW 264.7 cells (5 × 10^4^ cells/well) were grown under the same conditions for the cell proliferation activity assay. After the 24-h incubation, each well was added with FITC-labeled dextran (Sigma-Aldrich, St. Louis, MO, USA) and incubated for 30 min followed by discarding the cell culture and washing three times with PBS. The cells of each well were fully lysed with 200 μL Triton X-100 (1%) and the relative fluorescence units were measured by using the Synergy™ HTX Multi-Mode Microplate Reader (BioTek Instruments Inc., Winooski, VT, USA). Each treatment group was detected in triplicate, and three independent assays were performed.

### Determination of immune-related enzyme activity and cytokine level

RAW 264.7 monolayers (1 × 10^5^ cells/mL) were grown in 24-well plates, and stimulated with EcN_OMVs (1.0 μg/mL) and heated-killed EcN (the ratio of bacteria: cell = 25:1) for 16 h. After the incubation, the cell culture supernatant and cells from each well were harvested for determination of cytokines and immune-related enzymatic activities as follows: cytokines in the supernatant including IL-4, IL-6, IL-10, IL-12p40, and TNF-α; NO production in the supernatant; intracellular enzymatic activities including ACP and iNOS. Cytokine levels were determined by using the corresponding ELISA Kits (R&D System, Minneapolis, USA) according to the manufacturer’s protocol. NO production and enzymatic activities were determined by using the corresponding assay kit (Nanjing Jiancheng Bioengineering Institute, Jiangsu, China). Each treatment group was detected in triplicate, and three independent assays were performed.

### Antimicrobial activity assay

Three different bacterial pathogens were purchased from the China Veterinary Culture Collection Center (Beijing, China), including two Gram-negative species, *E. coli* CVCC1554 and *S. Typhimurium* CVCC3757, and one Gram-positive specie, *S. aureus* CVCC4265. Antimicrobial activity assay was performed as described previously [36]. In brief, RAW 264.7 monolayers (1 × 10^5^ cells/mL) were grown in 24-well plates and treated with EcN_OMVs (1.0 μg/mL) for 16 h followed by completely removing the supernatants and washing three times with PBS. The fresh antibiotic-free medium containing each bacterial pathogen was added to each well at a 100:1 bacteria/macrophage ratio. After incubation for 3 h at 37 °C, the cells were washed three times to remove non-adhered bacteria, and the fresh antibiotic-free medium was added. The cells were continuously incubated for 2 h (a total of 5 h of pathogen invasion). Subsequently, the cells were washed three times and then lysed with 1% TritonX-100 for 5 min at 37 °C. Cell lysates were immediately coated onto the corresponding agar plates for CFU determination. Each treatment had three replicates, and three independent assays were performed.

### Statistical analysis

All data were presented as mean ± SE. Student’s *t*-test was used for the analysis of differences between the two groups. One-way ANOVA analysis followed by Newman-Keuls’s multiple comparison test was used to compare the means among greater than two groups. Statistical significance was declared at *P* < 0.05. All data analyses were performed using Graph Pad Prism software 5.0 (San Diego, CA, USA).

## Supplementary information

**Additional file 1 Table S1.** EcN_OMVs proteins identified in this study

## Data Availability

All data analyzed during this study are included in this published article and its supplementary information files.

## Abbreviations

EcN: *Escherichia coli* strain nissle 1917
OMVs: Outer membrane vesicles
EVs: Extracellular vesicles
EcN_OMVs: Outer membrane vesicles secreted by *Escherichia coli* strain nissle 1917
NTA: Nanoparticle tracking analysis
COG: Clusters of orthologous groups of proteins
DiI: Dialkylcarbocyanine iodide
DAPI: 4, 6-diamidino-2-phenylindole
LDH: Lactic dehydrogenase
ACP: Acid phosphatase
iNOS: Inducible nitric oxide synthase
TNF-α: Tumor necrosis factor-alpha
IL-4: Interleukin-4
IL-6: Interleukin-6
IL-10: Interleukin-10
IL-12: Interleukin-12
IBD: Inflammatory bowel disease
LPS: Lipopolysaccharide
TLR: Toll-like receptor
NOD: Nod-like receptor
