# Supplementary material for: Probiotic Escherichia coli Nissle 1917-derived outer membrane vesicles enhance immunomodulation and antimicrobial activity in RAW264.7 macrophages
Source: BMC Microbiol. 2020 Aug 27;20:268. doi: 10.1186/s12866-020-01953-x (PMC7457259; doi:10.1186/s12866-020-01953-x)
Supplement: Supplementary file 1 — Additional file 1 Table S1. EcN_OMVs proteins identified in this study [file 12866_2020_1953_MOESM1_ESM.docx]

| **Table S1. EcN_OMVs proteins identiﬁed in this study.** | | | |
| --- | --- | --- | --- |
| **­Gene name** | **Protein accession** | **Protein description** | **GOG Functional Category** |
| **OuterMembrane** | | | |
| pta | P0A9M8 | Phosphate acetyltransferase | Energy production and conversion |
| envC | P37690 | Murein hydrolase activator EnvC | Cell cycle control, cell division, chromosome partitioning |
| lamB | P02943 | Maltoporin | Carbohydrate transport and metabolism |
| fbaB | P0A991 | Fructose-bisphosphate aldolase class 1 | Carbohydrate transport and metabolism |
| hemX | P09127 | Protein HemX | Coenzyme transport and metabolism |
| btuB | P06129 | Vitamin B12 transporter BtuB | Coenzyme transport and metabolism |
| fadL | P10384 | Long-chain fatty acid transport protein | Lipid transport and metabolism |
| bamE | P0A937 | Outer membrane protein assembly factor BamE | Translation, ribosomal structure and biogenesis |
| ydiY | P76206 | Uncharacterized protein YdiY | Cell wall/membrane/envelope biogenesis |
| focG | A0A0H2V658 | F1C minor fimbrial subunit protein G presursor | Cell wall/membrane/envelope biogenesis |
| tsx | P0A927 | Nucleoside-specific channel-forming protein Tsx | Cell wall/membrane/envelope biogenesis |
| tolC | P02930 | Outer membrane protein TolC | Cell wall/membrane/envelope biogenesis |
| tamA | P0ADE4 | Translocation and assembly module subunit TamA | Cell wall/membrane/envelope biogenesis |
| slp | P37194 | Outer membrane protein Slp | Cell wall/membrane/envelope biogenesis |
| pldA | P0A921 | Phospholipase A1 | Cell wall/membrane/envelope biogenesis |
| ompX | P0A917 | Outer membrane protein X | Cell wall/membrane/envelope biogenesis |
| ompW | P0A915 | Outer membrane protein W | Cell wall/membrane/envelope biogenesis |
| ompF | P02931 | Outer membrane protein F | Cell wall/membrane/envelope biogenesis |
| ompC | P06996 | Outer membrane protein C | Cell wall/membrane/envelope biogenesis |
| ompA | P0A910 | Outer membrane protein A | Cell wall/membrane/envelope biogenesis |
| focF | A8KIB2 | F1C minor fimbrial subunit F | Cell wall/membrane/envelope biogenesis |
| mltC | P0C066 | Membrane-bound lytic murein transglycosylase C | Cell wall/membrane/envelope biogenesis |
| mipA | P0A908 | MltA-interacting protein | Cell wall/membrane/envelope biogenesis |
| mepS | P0AFV4 | Murein DD-endopeptidase MepS/Murein LD-carboxypeptidase | Cell wall/membrane/envelope biogenesis |
| lptD | P31554 | LPS-assembly protein LptD | Cell wall/membrane/envelope biogenesis |
| lpp | P69776 | Major outer membrane prolipoprotein Lpp | Cell wall/membrane/envelope biogenesis |
| lolB | P61320 | Outer-membrane lipoprotein LolB | Cell wall/membrane/envelope biogenesis |
| lolA | P61316 | Outer-membrane lipoprotein carrier protein | Cell wall/membrane/envelope biogenesis |
| blc | P0A901 | Outer membrane lipoprotein Blc | Cell wall/membrane/envelope biogenesis |
| bamC | P0A903 | Outer membrane protein assembly factor BamC | Cell wall/membrane/envelope biogenesis |
| bamA | P0A940 | Outer membrane protein assembly factor BamA | Cell wall/membrane/envelope biogenesis |
| amiB | P26365 | N-acetylmuramoyl-L-alanine amidase AmiB | Cell wall/membrane/envelope biogenesis |
| focA | P0AC23 | Probable formate transporter 1 | Cell wall/membrane/envelope biogenesis |
| FocH | Q46686 | FocH protein | Cell wall/membrane/envelope biogenesis |
| fiu | P75780 | Catecholate siderophore receptor Fiu | Inorganic ion transport and metabolism |
| fhuA | P06971 | Ferrichrome outer membrane transporter/phage receptor | Inorganic ion transport and metabolism |
| fepA | P05825 | Ferrienterobactin receptor | Inorganic ion transport and metabolism |
| fecA | P13036 | Fe(3+) dicitrate transport protein FecA | Inorganic ion transport and metabolism |
| iutA | P14542 | Ferric aerobactin receptor | Inorganic ion transport and metabolism |
| yraP | P64596 | Uncharacterized protein YraP | General function prediction only |
| bcsC | P37650 | Cellulose synthase operon protein C | General function prediction only |
| bamD | P0AC02 | Outer membrane protein assembly factor BamD | General function prediction only |
| yncE | P76116 | Uncharacterized protein YncE | Function unknown |
| tamB | P39321 | Translocation and assembly module subunit TamB | Function unknown |
| pqiC | P0AB10 | Intermembrane transport lipoprotein PqiC | Function unknown |
| nanC/yjhA | P69856 | Probable N-acetylneuraminic acid outer membrane channel protein NanC | Function unknown |
| ygiM | P0ADT8 | Uncharacterized protein YgiM | Signal transduction mechanisms |
| tolC | P02930 | Outer membrane protein TolC | Intracellular trafficking, secretion, and vesicular transport |
| **Cytoplasmic** | | | |
| trxA | P0AA25 | Thioredoxin 1 | Energy production and conversion |
| sucC | P0A836 | Succinate--CoA ligase [ADP-forming] subunit beta | Energy production and conversion |
| sucA | P0AFG3 | 2-oxoglutarate dehydrogenase E1 component | Energy production and conversion |
| sdhB | P07014 | Succinate dehydrogenase iron-sulfur subunit | Energy production and conversion |
| sdhA | P0AC41 | Succinate dehydrogenase flavoprotein subunit | Energy production and conversion |
| pflB | P09373 | Formate acetyltransferase 1 | Energy production and conversion |
| nuoC | P33599 | NADH-quinone oxidoreductase subunit C/D | Energy production and conversion |
| narH | P11349 | Respiratory nitrate reductase 1 beta chain | Energy production and conversion |
| mdh | P61889 | Malate dehydrogenase | Energy production and conversion |
| maeB | P76558 | NADP-dependent malic enzyme | Energy production and conversion |
| lpdA | P0A9P0 | Dihydrolipoyl dehydrogenase | Energy production and conversion |
| glpK | P0A6F3 | Glycerol kinase | Energy production and conversion |
| glpD | P13035 | Aerobic glycerol-3-phosphate dehydrogenase | Energy production and conversion |
| frdA | P00363 | Fumarate reductase flavoprotein subunit | Energy production and conversion |
| atpD | P0ABB4 | ATP synthase subunit beta | Energy production and conversion |
| atpA | P0ABB0 | ATP synthase subunit alpha | Energy production and conversion |
| adhE | P0A9Q7 | Aldehyde-alcohol dehydrogenase | Energy production and conversion |
| aceF | P06959 | Dihydrolipoyllysine-residue acetyltransferase component of pyruvate dehydrogenase complex | Energy production and conversion |
| mreB | P0A9X4 | Cell shape-determining protein MreB | Cell cycle control, cell division, chromosome partitioning |
| minD | P0AEZ3 | Septum site-determining protein MinD | Cell cycle control, cell division, chromosome partitioning |
| tnaA | P0A853 | Tryptophanase | Amino acid transport and metabolism |
| prs | P0A717 | Ribose-phosphate pyrophosphokinase | Amino acid transport and metabolism |
| pyrG | P0A7E5 | CTP synthase | Nucleotide transport and metabolism |
| prs | P0A717 | Ribose-phosphate pyrophosphokinase | Nucleotide transport and metabolism |
| rbsA | P04983 | Ribose import ATP-binding protein RbsA | Carbohydrate transport and metabolism |
| pgk | P0A799 | Phosphoglycerate kinase | Carbohydrate transport and metabolism |
| gapA | P0A9B2 | Glyceraldehyde-3-phosphate dehydrogenase A | Carbohydrate transport and metabolism |
| eno | P0A6P9 | Enolase | Carbohydrate transport and metabolism |
| crr | P69783 | PTS system glucose-specific EIIA component | Carbohydrate transport and metabolism |
| fabZ | P0A6Q6 | 3-hydroxyacyl-[acyl-carrier-protein] dehydratase FabZ | Lipid transport and metabolism |
| fabB | P0A953 | 3-oxoacyl-[acyl-carrier-protein] synthase 1 | Lipid transport and metabolism |
| acpP | P0A6A8 | Acyl carrier protein | Lipid transport and metabolism |
| accA | P0ABD5 | Acetyl-coenzyme A carboxylase carboxyl transferase subunit alpha | Lipid transport and metabolism |
| tufB | P0CE48 | Elongation factor Tu 2 | Translation, ribosomal structure and biogenesis |
| tsf | P0A6P1 | Elongation factor Ts | Translation, ribosomal structure and biogenesis |
| rpsU | P68679 | 30S ribosomal protein S21 | Translation, ribosomal structure and biogenesis |
| rpsN | P0AG59 | 30S ribosomal protein S14 | Translation, ribosomal structure and biogenesis |
| rpsL | P0A7S3 | 30S ribosomal protein S12 | Translation, ribosomal structure and biogenesis |
| rpsK | P0A7R9 | 30S ribosomal protein S11 | Translation, ribosomal structure and biogenesis |
| rpsJ | P0A7R5 | 30S ribosomal protein S10 | Translation, ribosomal structure and biogenesis |
| rpsI | P0A7X3 | 30S ribosomal protein S9 | Translation, ribosomal structure and biogenesis |
| rpsH | P0A7W7 | 30S ribosomal protein S8 | Translation, ribosomal structure and biogenesis |
| rpsG | P02359 | 30S ribosomal protein S7 | Translation, ribosomal structure and biogenesis |
| rpsE | P0A7W1 | 30S ribosomal protein S5 | Translation, ribosomal structure and biogenesis |
| rpsB | P0A7V0 | 30S ribosomal protein S2 | Translation, ribosomal structure and biogenesis |
| rplV | P61175 | 50S ribosomal protein L22 | Translation, ribosomal structure and biogenesis |
| rplS | P0A7K6 | 50S ribosomal protein L19 | Translation, ribosomal structure and biogenesis |
| rplQ | P0AG44 | 50S ribosomal protein L17 | Translation, ribosomal structure and biogenesis |
| rplO | P02413 | 50S ribosomal protein L15 | Translation, ribosomal structure and biogenesis |
| rplL | P0A7K2 | 50S ribosomal protein L7/L12 | Translation, ribosomal structure and biogenesis |
| rplF | P0AG55 | 50S ribosomal protein L6 | Translation, ribosomal structure and biogenesis |
| rplD | P60723 | 50S ribosomal protein L4 | Translation, ribosomal structure and biogenesis |
| rplC | P60438 | 50S ribosomal protein L3 | Translation, ribosomal structure and biogenesis |
| rpoA | P0A7Z4 | DNA-directed RNA polymerase subunit alpha | Transcription |
| ygeH | P76639 | Uncharacterized protein YgeH | Transcription |
| rpoB | P0A8V2 | DNA-directed RNA polymerase subunit beta | Transcription |
| prc | P23865 | Tail-specific protease | Cell wall/membrane/envelope biogenesis |
| kdsA | P0A715 | 2-dehydro-3-deoxyphosphooctonate aldolase | Cell wall/membrane/envelope biogenesis |
| amiA | P36548 | N-acetylmuramoyl-L-alanine amidase AmiA | Cell wall/membrane/envelope biogenesis |
| trxA | P0AA25 | Thioredoxin 1 | Posttranslational modification, protein turnover, chaperones |
| tpx | P0A862 | Thiol peroxidase | Posttranslational modification, protein turnover, chaperones |
| hflC | P0ABC3 | Modulator of FtsH protease HflC | Posttranslational modification, protein turnover, chaperones |
| groL | P0A6F5 | 60 kDa chaperonin | Posttranslational modification, protein turnover, chaperones |
| ftnA | P0A998 | Bacterial non-heme ferritin | Inorganic ion transport and metabolism |
| dps | P0ABT2 | DNA protection during starvation protein | Inorganic ion transport and metabolism |
| bfr | P0ABD3 | Bacterioferritin | Inorganic ion transport and metabolism |
| fabB | P0A953 | 3-oxoacyl-[acyl-carrier-protein] synthase 1 | Secondary metabolites biosynthesis, transport and catabolism |
| acpP | P0A6A8 | Acyl carrier protein | Secondary metabolites biosynthesis, transport and catabolism |
| ycfL | P75946 | Uncharacterized protein YcfL | General function prediction only |
| grcA | P68066 | Autonomous glycyl radical cofactor | General function prediction only |
| yhcB | P0ADW3 | Inner membrane protein YhcB | Function unknown |
| elaB | P0AEH5 | Protein ElaB | Function unknown |
| **Periplasmic** | | | |
| glpQ | P09394 | """Glycerophosphodiester phosphodiesterase, periplasmic""" | Energy production and conversion |
| ftsN | P29131 | Cell division protein FtsN | Cell cycle control, cell division, chromosome partitioning |
| proX | P0AFM2 | Glycine betaine/proline betaine-binding periplasmic protein | Amino acid transport and metabolism |
| oppA | P23843 | Periplasmic oligopeptide-binding protein | Amino acid transport and metabolism |
| hisJ | P0AEU0 | Histidine-binding periplasmic protein | Amino acid transport and metabolism |
| gltI | P37902 | Glutamate/aspartate import solute-binding protein | Amino acid transport and metabolism |
| glnH | P0AEQ3 | Glutamine-binding periplasmic protein | Amino acid transport and metabolism |
| fliY | P0AEM9 | L-cystine-binding protein FliY | Amino acid transport and metabolism |
| artI | P30859 | Putative ABC transporter arginine-binding protein 2 | Amino acid transport and metabolism |
| ansB | P00805 | L-asparaginase 2 | Amino acid transport and metabolism |
| cpdB | P08331 | """2',3'-cyclic-nucleotide 2'-phosphodiesterase/3'-nucleotidase""" | Nucleotide transport and metabolism |
| mglB | P0AEE5 | D-galactose-binding periplasmic protein | Carbohydrate transport and metabolism |
| malE | P0AEX9 | Maltose/maltodextrin-binding periplasmic protein | Carbohydrate transport and metabolism |
| ansB | P00805 | L-asparaginase 2 | Translation, ribosomal structure and biogenesis |
| skp | P0AEU7 | Chaperone protein Skp | Cell wall/membrane/envelope biogenesis |
| lptE | P0ADC1 | LPS-assembly lipoprotein LptE | Cell wall/membrane/envelope biogenesis |
| emtA | P0C960 | Endo-type membrane-bound lytic murein transglycosylase A | Cell wall/membrane/envelope biogenesis |
| amiC | P63883 | N-acetylmuramoyl-L-alanine amidase AmiC | Cell wall/membrane/envelope biogenesis |
| flgI | P0A6S3 | Flagellar P-ring protein | Cell motility |
| flgA | P75933 | Flagella basal body P-ring formation protein FlgA | Cell motility |
| ppiA | P0AFL3 | Peptidyl-prolyl cis-trans isomerase A | Posttranslational modification, protein turnover, chaperones |
| fkpA | P45523 | FKBP-type peptidyl-prolyl cis-trans isomerase FkpA | Posttranslational modification, protein turnover, chaperones |
| dsbC | P0AEG6 | Thiol:disulfide interchange protein DsbC | Posttranslational modification, protein turnover, chaperones |
| degP | P0C0V0 | Periplasmic serine endoprotease DegP | Posttranslational modification, protein turnover, chaperones |
| metQ | P28635 | D-methionine-binding lipoprotein MetQ | Inorganic ion transport and metabolism |
| mdoD | P40120 | Glucans biosynthesis protein D | Inorganic ion transport and metabolism |
| mlaC | P0ADV7 | Intermembrane phospholipid transport system binding protein MlaC | Secondary metabolites biosynthesis, transport and catabolism |
| osmY | P0AFH8 | Osmotically-inducible protein Y | General function prediction only |
| lpoA | P45464 | Penicillin-binding protein activator LpoA | General function prediction only |
| ybiS | P0AAX8 | """Probable L,D-transpeptidase YbiS""" | Function unknown |
| cpoB | P45955 | Cell division coordinator CpoB | Function unknown |
| ybaY | P77717 | Uncharacterized lipoprotein YbaY | Function unknown |
| hisJ | P0AEU0 | Histidine-binding periplasmic protein | Signal transduction mechanisms |
| gltI | P37902 | Glutamate/aspartate import solute-binding protein | Signal transduction mechanisms |
| glnH | P0AEQ3 | Glutamine-binding periplasmic protein | Signal transduction mechanisms |
| fliY | P0AEM9 | L-cystine-binding protein FliY | Signal transduction mechanisms |
| artI | P30859 | Putative ABC transporter arginine-binding protein 2 | Signal transduction mechanisms |
| tolB | P0A855 | Tol-Pal system protein TolB | Intracellular trafficking, secretion, and vesicular transport |
| amiD | P75820 | N-acetylmuramoyl-L-alanine amidase AmiD | Defense mechanisms |
| ampH | P0AD70 | D-alanyl-D-alanine-carboxypeptidase/endopeptidase AmpH | Defense mechanisms |
| ampC | P00811 | Beta-lactamase | Defense mechanisms |
| **InnerMembrane** | | | |
| cyoA | P0ABJ1 | Cytochrome bo(3) ubiquinol oxidase subunit 2 | Energy production and conversion |
| cydA | P0ABJ9 | Cytochrome bd-I ubiquinol oxidase subunit 1 | Energy production and conversion |
| ptsG | P69786 | PTS system glucose-specific EIICB component | Carbohydrate transport and metabolism |
| manZ | P69805 | PTS system mannose-specific EIID component | Carbohydrate transport and metabolism |
| mrcB | P02919 | Penicillin-binding protein 1B | Cell wall/membrane/envelope biogenesis |
| fliP | P0AC05 | Flagellar biosynthetic protein FliP | Cell motility |
| ftsH | P0AAI3 | ATP-dependent zinc metalloprotease FtsH | Posttranslational modification, protein turnover, chaperones |
| fecB | P15028 | Fe(3+) dicitrate-binding periplasmic protein | Inorganic ion transport and metabolism |
| eptA | P30845 | Phosphoethanolamine transferase EptA | General function prediction only |
| elyC | P0AB01 | Envelope biogenesis factor ElyC | Function unknown |
| yidC | P25714 | Membrane protein insertase YidC | Intracellular trafficking, secretion, and vesicular transport |
| yajC | P0ADZ7 | Sec translocon accessory complex subunit YajC | Intracellular trafficking, secretion, and vesicular transport |
| tolR | P0ABV6 | Tol-Pal system protein TolR | Intracellular trafficking, secretion, and vesicular transport |
| tolQ | P0ABU9 | Tol-Pal system protein TolQ | Intracellular trafficking, secretion, and vesicular transport |
| secD | P0AG90 | Protein translocase subunit SecD | Intracellular trafficking, secretion, and vesicular transport |
| lepB | P00803 | Signal peptidase I | Intracellular trafficking, secretion, and vesicular transport |
| fliP | P0AC05 | Flagellar biosynthetic protein FliP | Intracellular trafficking, secretion, and vesicular transport |
| acrB | P31224 | Multidrug efflux pump subunit AcrB | Defense mechanisms |
| msbA | P60752 | Lipid A export ATP-binding/permease protein MsbA | Defense mechanisms |
| **Extracellular** | | | |
| ybhC | P46130 | Putative acyl-CoA thioester hydrolase YbhC | Carbohydrate transport and metabolism |
| rlpA | P10100 | Endolytic peptidoglycan transglycosylase RlpA | Cell wall/membrane/envelope biogenesis |
| pal | P0A912 | Peptidoglycan-associated lipoprotein | Cell wall/membrane/envelope biogenesis |
| nlpD | P0ADA3 | Murein hydrolase activator NlpD | Cell wall/membrane/envelope biogenesis |
| mltA | P0A935 | Membrane-bound lytic murein transglycosylase A | Cell wall/membrane/envelope biogenesis |
| fliC | P04949 | Flagellin | Cell motility |
| flgL | P29744 | Flagellar hook-associated protein 3 | Cell motility |
| flgK | P33235 | Flagellar hook-associated protein 1 | Cell motility |
| flgH | P0A6S0 | Flagellar L-ring protein | Cell motility |
| flgG | P0ABX5 | Flagellar basal-body rod protein FlgG | Cell motility |
| flgE | P75937 | Flagellar hook protein FlgE | Cell motility |
